# AKT/eNOS signaling module functions as a potential feedback loop in the growth hormone signaling pathway

**DOI:** 10.1186/1750-2187-4-1

**Published:** 2009-03-25

**Authors:** Cong-Jun Li, Theodore H Elsasser, Stanislaw Kahl

**Affiliations:** 1 Bovine Functional Genomics laboratory, Animal and Natural Resources Institute, Agricultural Research Service, US Department of Agriculture, 10300 Baltimore Ave., BARC EAST, Building 200, Room 209, Beltsville, MD 20705, USA

## Abstract

**Background:**

While evidence suggested that the activity states of Protein kinase B (AKT/PKB) and endothelial nitric oxide synthase (eNOS) play an important role in the progression of the Growth Hormone (GH) signal cascade, the implication of the activation of AKT/PKB and eNOS in terms of their function in the signaling pathway was not clear.

**Results:**

Using a specific AKT/PKB inhibitor and a functional proteomic approach, we were able to detect the activities of multiple signal transduction pathway elements, the downstream targets of the AKT/PKB pathway and the modification of those responses by treatment with GH. Inhibiting the AKT/PKB activity reduced or eliminated the activation (phosphorylation) of eNOS. We demonstrated that the progression of the GH signal cascade is influenced by the activity status of AKT and eNOS, wherein the suppression of AKT activity appears to augment the activity of extracellular signal-regulated kinases 1 and 2 (Erk1/2) and to antagonize the deactivation (phosphorylation) of cyclin-dependent kinase 2 (CDC2/Cdk1) induced by GH. Phosphorylation of GSK3a/b (glycogen synthase kinase 3), the downstream target of AKT/PKB, was inhibited by the AKT/PKB inhibitor. GH did not increase phosphorylation of ribosomal S6 kinase 1 (RSK1) in normal cells but increases phosphorylation of RSK1 in cells pre-treated with the AKT and eNOS inhibitors.

**Conclusion:**

The MAP kinase and CDC2 kinase-dependent intracellular mechanisms are involved in or are the targets of the GH's action processes, and these activities are probably directly or indirectly modulated by AKT/PKB pathways. We propose that the AKT/PKB-eNOS module likely functions as a negative feedback mediator of GH actions.

## Background

Endothelial nitric oxide synthase (eNOS or NOS3) is a target downstream of activated AKT/PKB. In response to various forms of cellular stimulation, eNOS is phosphorylated by AKT/PKB [1]. The AKT proto-oncogene is an important regulator of various cellular processes, including glucose metabolism and cell survival [2-5]. AKT/PKB can be phosphorylated and activated by the activation of receptor tyrosine kinases and the G-protein-coupled receptor, as well as the stimulation of cells by mechanical forces [6]. The relationships between the activation of AKT, its downstream effectors and the production of soluble second messengers, have been studied extensively, and comprehensive insight into the regulation and function of AKT has been gained over the past ten years [7]. It has become increasingly evident that the defects in AKT/PKB signaling also underlie a diverse array of diseases. Therefore, the clarification of AKT's downstream targets and functions is of central importance to our understanding and treatment of such diseases.

Exogenous growth hormone (GH) treatment has been explored as one potential adjunct for managing the catabolic processes that challenge the host response to infection stress [8,9]. Few definitive studies in cattle, if any, have addressed NO production as affected by GH treatment prior to the onset of immune challenge as well as signal transduction pathways that involved. The effects of GH are modulated through coordinated changes in gene expression and/or the function of the gene products by phosphorylation or other modifications that are the outcome of interactions between hormone-activated signal transduction pathways and specific feedback loops [10]. Coordinated cellular responses to GH are subsequently modulated through a molecular cascade involving both the activation and activation-termination of post-receptor signal transduction element responses [11,12]. GH actions at the cellular level can also be thought of in terms of the reactivity of tissues changing between "GH responsive" and "GH resistant" states [13,14]. These changing states of relative GH responsiveness have come to be recognized as vital for the establishment of such diverse attributes as the sexual dimorphic character of GH responsiveness [15], short-term GH-derived refractoriness [16] and the reprioritization of GH-directed actions on metabolism that develops during infection [17,18].

Studies from our laboratory have suggested that the production of nitrated protein during low-level, pro-inflammation stress, is increased by GH treatment through a direct effect on the competing activities of NOS and arginase, modulating critical control points in the proinflammatory cascade [17]. More recently, we also demonstrated that tyrosine residues 1007 and 1008 of Janus kinase 2 (JAK2), which are critical to the GH/cytokine receptor phosphorylation activation of JAK2 [19], could become nitrated [20]. This particular nitration developed in and was co-localized to membrane caveolae and their accompanying content of eNOS, and the recently characterized caveolar localization of the GH receptor [21,22].

While evidence suggested that the activity states of AKT and eNOS play an important role in the progression of the GH signal cascade [23-25], the implication of the activation of AKT/PKB and eNOS in terms of their function in the signaling pathway was not clear. Our hypothesis is that the GH-mediated activation of the AKT/PKB and eNOS signaling module functions not only as a positive affecter, but also as a negative feedback mediator of GH actions, that can, with specificity, affect and coordinate GH signal transduction processes in cells. The purpose of the present study was to examine *in vitro *responses of the potential signal transduction pathway elements of bovine cells and the modification of this response by treatment with recombinant GH. Using specific AKT/PKB and eNOS inhibitors and a functional proteomic approach *in vitro*, we were able to detect the activities of multiple potential signal transduction pathway elements; the downstream targets of AKT pathway; and the modification of those responses by treatment with GH. Combining the data from the *in vitro *experiments with the functional proteomic analysis, we propose that the AKT/eNOS signaling module may function as a feedback loop in the GH signaling pathway.

## Results

### The AKT/PKB inhibitor effectively inhibited phosphorylation (activation) of AKT, and eNOS

**Figure 1:**
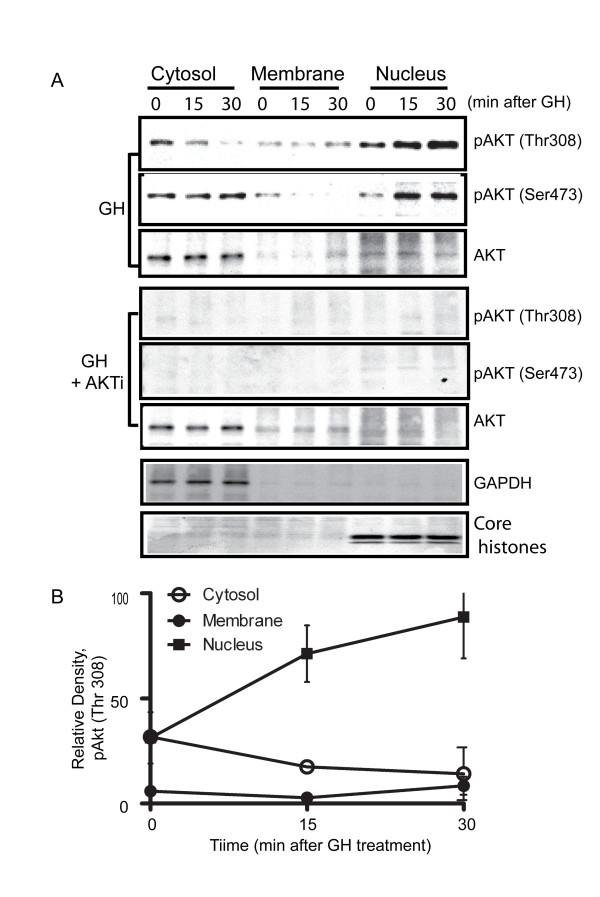
**Panel A: Cells with or without pre-treatment of AKT inhibitor (AKTi) were challenged with GH at 200 ng/ml for 0, 15 and 30 min respectively**. Cellular proteins were extracted from MDBK cells using the ProteoExtract^® ^subcellular extraction kit (Calbiochem) following the manufacturer's instructions. Western blotting experiments were conducted and analyzed as specified in Material and Methods. Panel B shows the quantitation of AKT phosphorylation (p-Thr308) after GH treatment.

**Figure 2:**
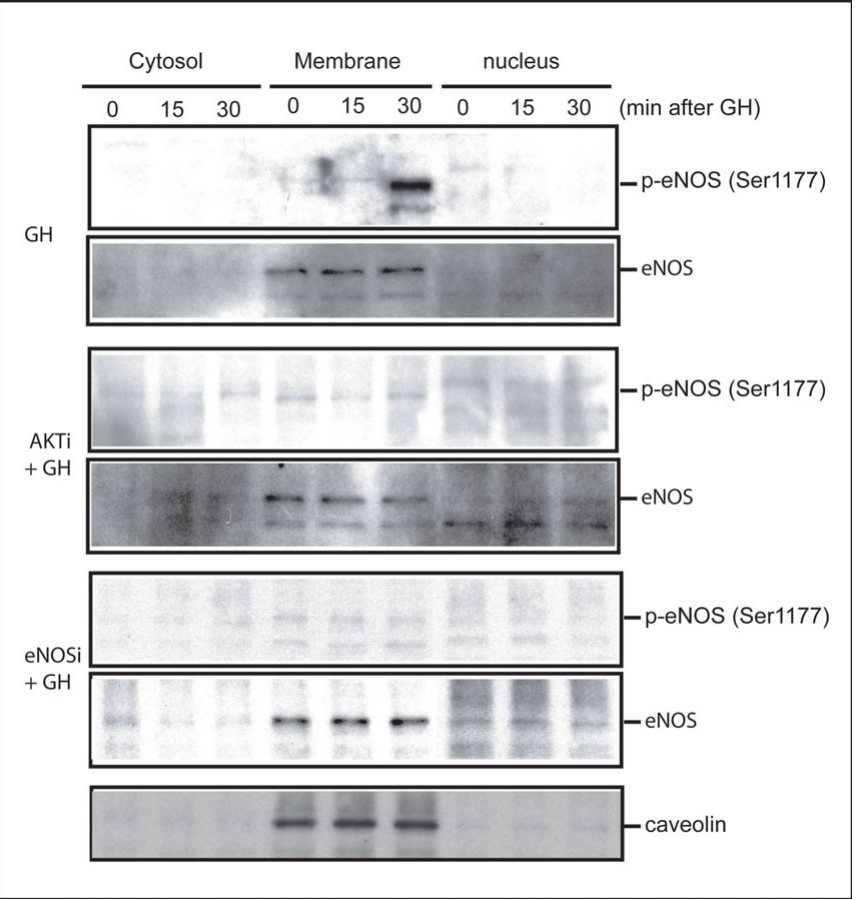
**AKT inhibitor eliminates the phosphorylation of eNOS (Ser1177)**. MDBK cells with or without pre-treatment of AKT inhibitor for 30 min were challenged with GH at 200 ng/ml for 0. 15 and 30 min respectively. At the indicated time points, proteins were extracted from MDBK cells using the ProteoExtract^® ^subcellular extraction kit (Calbiochem) and Western blot analyses were performed. Caveolin was used as a membrane protein marker. AKTi: AKT inhibitor. AKTi + GH: Cells pretreated with AKT inhibitor for 30 min before GH administration.

The MDBK cell culture system was used as a model to examine whether the function of AKT/PKB may be associated with the ability of GH to modulate the activation state of the NO-generating enzyme eNOS. Akt (protein kinase B), a subfamily of the AGC serine/threonine kinases, plays critical roles in survival, proliferation, glucose metabolism, and other cellular functions. Akt activation requires the recruitment of the enzyme to the plasma membrane by interacting with membrane-bound lipid products of phosphatidylinositol 3-kinase [26]. Membrane-bound Akt is then phosphorylated at two sites for its full activation [27]; Thr-308 in the activation loop of the kinase domain is phosphorylated by 3-phosphoinositide-dependent kinase-1 (PDK1) and Ser-473 in the C-terminal hydrophobic motif by a putative kinase PDK2. In order to determine whether the intracellular generation of p-eNOS and p-AKT by GH could be localized to a given compartment, subcellular fractionation was used. Proteins were extracted from MDBK cells using the ProteoExtract^® ^subcellular extraction kit (Calbiochem). We preconditioned MDBK cells in media containing AKT inhibitors for 30 min and investigated the effect of blocking eNOS phosphorylation after GH treatment. First, after treatment with GH (Figure 1A), Western blots for AKT, pThr308-AKT and p-Ser473-AKT clearly indicated that GH increased the intensity band for p-AKT (Thr308 and Ser473) (Figure 1A). Both pThr308-AKT and p-Ser473-AKT increased the total amount of phosphorylation, with pThr308 decreasing slightly in cytosol. Since AKT/PKB is activated by phosphorylation at Thr308 and Ser473, we can safely assume that the GH challenge stimulated AKT/PKB activity. Most of the phosphorylated AKT was localized in the nuclear fraction. Although phosphorylation of AKT in cytosol fraction shows a slight decrease in density, overall the trend of total phosphorylation of AKT from three cellular fractions does not change. We suspect that this discrepancy may be the result of the trans-location of the pAKT protein after GH stimulation. We also noted that there is a slight difference in the subcellular localization of AKT phosphorylated at Thr308 and Ser473 (Figure 1, panel A). This difference is consistent in replicated experiments. We do not have explanation for this observation. However, since two phosphorylation sites (Ser473 and Thr308) are phosphorylated by two different kinases and phosphorylation of two sites is not dependent to each other [27], we speculate the possibility that two populations of AKT, one phosphorylated at Thr308 and the other at Ser473 may exist. Pre-treatment of cells with AKT inhibitor for 30 minutes, however, eliminated the phosphorylation of AKT at both sites. Moreover, in parallel, Western blotting experiments using the same subcellular fractions were conducted to analyze the phosphorylation of eNOS, a membrane bound enzyme. Our data show that, after treatment GH, eNOS phosphorylation at Ser1177 was elevated 30 minutes after the GH challenge and phosphorylation of eNOS at Ser1177 is AKT-dependent (Figure 2). AKT can directly phosphorylated eNOS at Ser1177 and activate the enzyme [1,28,29]. When cells were pre-treated with the AKT inhibitor, the specific generation of phosphorylated eNOS at Ser1177 decreased to almost undetectable levels. Since eNOS phosphorylation by AKT could be direct or indirect, eNOS inhibitor (1 mM N^G^-Monomethyl arginine, CalBiochem) was also used in this experiment as a control to test actual eNOS dependency (Figure 2). Both phosphorylated eNOS and eNOS were localized in the membrane fraction (Figure 2) as indicated by caveolin, a membrane protein marker. This is consistent with our previous observation *in vivo *that phosphorylated eNOS is caveolae-associated [30]. In addition to the localization of eNOS, we also see that more than 90% of histone appears in the fraction of the nucleus (Figure 1A), and GAPDH appears only in the cytosol fraction (Figure 1A). All of these data indicated that the subcellular extraction procedure produced clean cellular fractionations.

**Figure 3:**
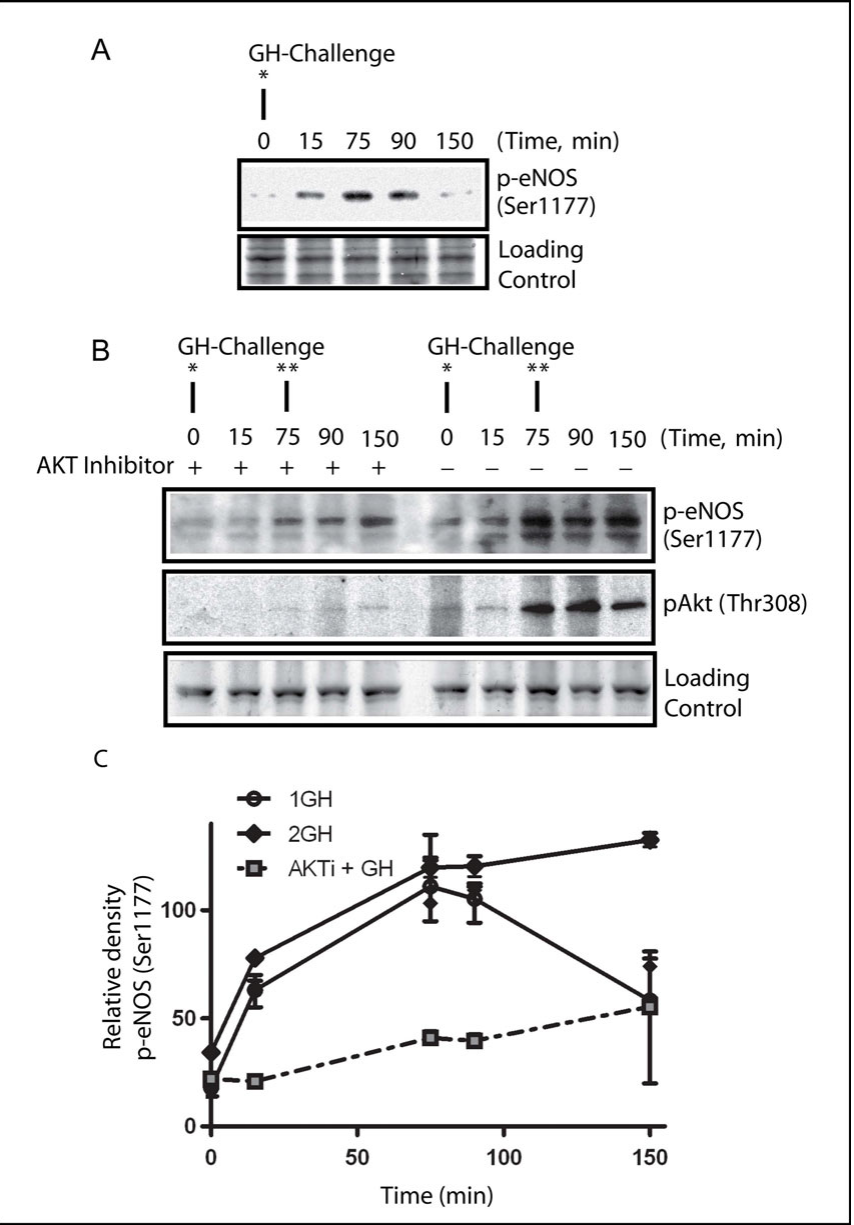
**The changes in the phosphorylation of eNOS after one or two GH challenges**. GH was administrated first at 0 min and again time at 75 min to MDBK cell cultures with or without AKT inhibitor pre-treatment. Whole cell extracts were prepared at the time point 0, 15, 75, 90 and 150 min after first GH challenge. Western blots using the antibodies against phospho-eNOS and phospho-AKT were performed. Panel A: The eNOS phosphorylation after one GH challenge. Panel B: The eNOS phosphorylation with two GH challenges, with or without AKT inhibitor pre-treatment for 30 min. Western blot with phospho-AKT (Thr308) was used to monitor the effectiveness of the AKT inhibitor. Loading control: Identical gel stained with SimplyBlue™ (Invitrogen). Panel C shows the quantitation of eNOS phosphorylation (Ser1177) after GH treatment. 1 GH: the quantitation for one GH challenge; 2 GH: quantitation for two GH challenge; AKTi + GH: AKT inhibitor pretreated for 30 min before GH challenge; *: First GH challenge, **: second GH challenge.

The metabolic effects of GH are either chronic diabetogenic or acute insulin-like. After exposure to GH the ability of the cells to respond with insulin-like effects disappears within one to three hours [10,31]. We suspect that AKT/eNOS may function as the negative feedback loop and play an important role in GH-induced GH desensitization. Therefore, we next performed a kinetic experiment to assess the changes in the phosphorylation of eNOS over the time course of GH stimulation, as well as the response of eNOS to secondary GH stimulation. GH was administrated at 0 min and followed with a second treatment at 75 min to normal cultured cells and cells preconditioned with the AKT inhibitor. The Western blot data (Figure 3) indicated that the GH-induced phosphorylation of eNOS started about 15 minutes after GH treatment and the phosphorylation of eNOS was saturated at about 75 min. In order to further investigate whether the cells retained their sensitivity to GH at the peak of the reaction to the first stimulation, as measured by the phosphorylation of eNOS, we administrated a second GH stimulation at this time point (75 min post first GH). Notably, the phosphorylation of eNOS levelled off after 75 minutes, and secondary stimulation with GH did not induce further phosphorylation of eNOS; rather, the level of eNOS phosphorylation was maintained. On the other hand, the AKT inhibitor significantly (P < 0.01) reduced the level of eNOS phosphorylation and therefore almost eliminated the activation of eNOS.

**Figure 4:**
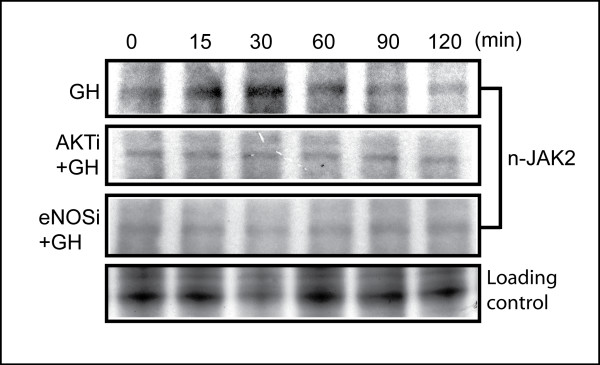
**Both AKT and eNOS inhibitors eliminate the nitration of JAK2**. GH was administrated to cell cultures with or without either AKT or eNOS inhibitor pre-treatment for 30 min. Whole cell extracts were analyzed with Western blot using an antibody (Anti-nitro-_1007_-Y_1008_Y-JAK2). AKTi: AKT inhibitor, eNOSi: eNOS inhibitor. Loading control: Identical gel stained with SimplyBlue™ (Invitrogen).

Since the GH-mediated nitration of JAK2 in critical regulatory tyrosine-associated sites has been found to serve as a novel regulatory post-translational modification, complementary but antagonistic to phosphorylation [20,30], we investigated the effects of the AKT and eNOS inhibitors on the nitration of JAK-2 (nitro-JAK2) in MDBK cells with a specific antibody for nitrated JAK2 [20]. Normal growing cells and cells pre-treated with the AKT and eNOS inhibitors were treated with GH at 200 ng/ml. Proteins were extracted at 0, 15, 30, 60, 90 and 120 min. Western blots were performed with anti-nitro-JAK2. Fifteen minutes after treatment with GH, JAK2 was nitrated, with the highest level observed at about 30 minutes (Figure 4). In contrast, at both the 15 minute and the 30 minute time points, the specific generation of the nitrated JAK2 decreased after GH when the cells were pre-treated with either the AKT or eNOS inhibitor. Therefore, this experiment and the temporal patterns of JAK2 nitration confirmed that both AKT and eNOS are functioning as regulators of JAK2 nitration [17,30].

### Downstream elements of cellular signal transduction pathways affected by GH and pre-treatment with the AKT inhibitor

**Figure 5:**
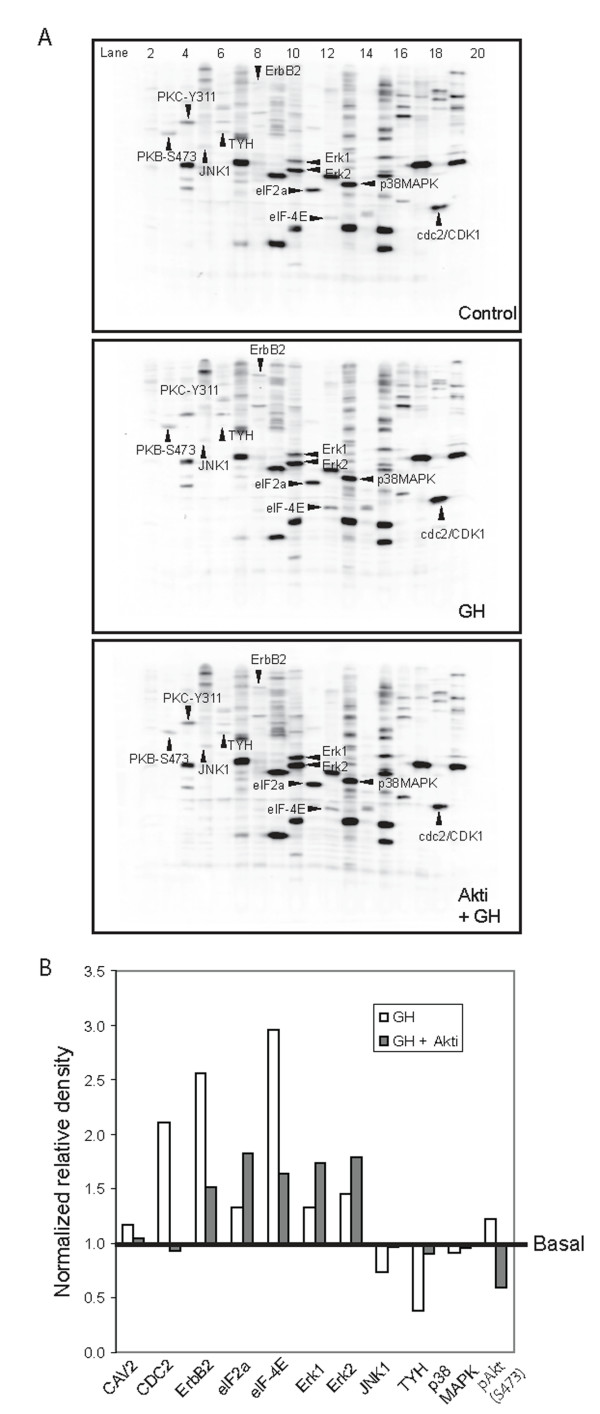
**Panel A: KPSS-6.0 phosphoantibody multi-immunoblot array; MDBK cells were treated with or without the AKT inhibitor for 30 minutes before GH administration**. The whole cell lysates were prepared after 30 min of GH challenge and analyzed using the KPSS-6.0 phosphoantibody multi-immunoblot array by Kinexus . The phosphor-protein targets of interest are indicated by arrows and abbreviations. The upper panel gives the array analysis of normal cells without GH (control); the middle panel shows the array analysis of GH treated cells; the lower panel gives the array analysis of the AKT inhibitor pretreated cells before GH administration. Panel B: Normalized quantitation of the results of KPSS-6.0 phosphoantibody multi-immunoblot array. The quantitation is the trace quantity of the band corrected to a scan time of 60 seconds. The normalized quantitation is the quantitation normalized to correct for differences in protein amounts.

To further investigate the roles of AKT/PKB in GH signaling pathways, we utilized a global proteomics phospho-antibody array-based approach (Kinetworks Phospho-Site Screen (KPSS)-6.0, ) to analyze the signal transduction pathways of the GH axis and the roles of AKT/PKB in Madin-Darby bovine kidney epithelial (MDBK) cells. Samples were prepared according to the instruction of the service provider, and sent to the service provider for phosphor-site screen and quantitation. Essentially, the entire cell lysate sample (an equal amount of protein from each of sample) was applied in one lane that spanned the width of the SDS-PAGE gel. A multi-blotting apparatus that features 20 separate slot chambers was used for incubation with multiple primary antibodies and then secondary antibodies. The immunoblot was incubated with an enhanced chemo-luminescence ECL-plus reagent. The ECL signal produced on the immunoblot was detected using a Bio-Rad FluorS Max Multi-Imager with a 16-bit camera and with the Bio-Rad Quantity One software program for quantitation. The band densities were normalized to correct for differences in the amounts of protein. The normalized data were obtained from the service provider. A diverse set of phospho-proteins pivotal in a wide range of cellular regulations were identified (Figure 5A) and quantitated (Figure 5B). Remarkably, CDC2/Cdk1 (Cell division control protein 2/Cyclin-dependent protein kinase 1) became hyperphosphorylated (deactivated) at 30 minutes after GH treatment, whereas pre-treatment of cells with the AKT inhibitor antagonized the deactivation of CDC2/Cdk1 induced by GH. Since the data were normalized to correct for the differences in amounts of protein, the quantitation reflects the relative levels of CDC2/Cdk1 protein phosphorylation. The AKT inhibitor antagonized the effects of GH on the stress-activated proteins kinase (cJun NH2 terminal kinase, JNK1) and tyrosine hydroxylase (TYH), and restored the phosphorylation level of both proteins. Pre-treatment with the AKT inhibitor also inhibited the phosphorylation of the ErbB2 receptor tyrosine kinase (ErbB2) and eukaryotic translation initiation factor 4 (elF-4E). On the other hand, AKT inhibitor augmented the already increased activities of the translation initiation factor elF2a, extracellular signal-regulated kinases Erk1 and Erk2 after GH treatment by stimulating the additional phosphorylation of elF2a, Erk1 and Erk2. The data also showed that the AKT inhibitor reduced the phosphorylation of AKT to lower than 40% of the basal level. We noted that the basal levels of some proteins such as ERK1/2 and AKT were substantial in this particular experiment. We suspect that this was due to the cell's sensibility to manipulation, such as the stress of aspirating and replacing the medium-containing vehicle for the control cells. The substantial basal levels seem not affecting our conclusion, however, it may decrease the ability to detect more GH-dependent events. Therefore appropriate precaution should be employed in future experiments.

**Figure 6:**
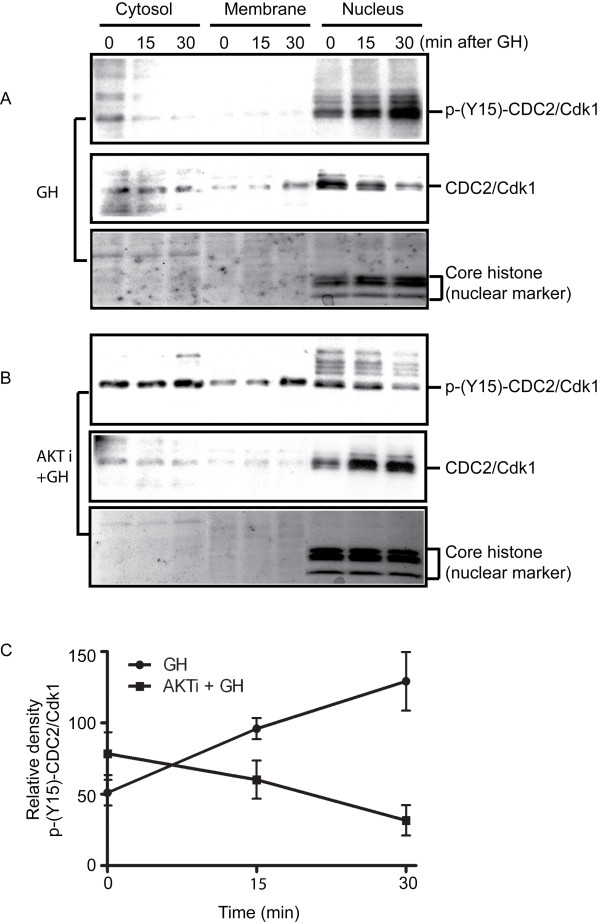
**Phosphorylation states of CDC2 in a time course of GH treatment in the cell culture with or without AKT inhibitor (AKTi) pre-treatment**. After treatment with GH at the indicated time points, proteins were extracted from MDBK cells using the ProteoExtract^® ^subcellular extraction kit (Calbiochem). Two identical gels were transferred to nitrocellulose membrane and Western blot analyses were performed for total CDC2/Cdk1 and phosphor-CDC2/Cdk1. Panel A: Temporal changes of phospho-CDC2/Cdk1 (p-(Y15)-CDC2/Cdk1 and total CDC2/Cdk1 after administration of GH. Panel B: Temporal changes of phosphor-CDC2/Cdk1 (p-(Y15)-CDC2/Ckd1) and total CDC2/Cdk1 in AKT inhibitor (AKTi)-pretreated MDBK cells after administration of GH. Panel C: Quantitation of phosphor-CDC2/Cdk1 (p-(Y15)-CDC2/Cdk1) from panels A and B. n = 2 replicated experiments.

GH is an important endocrine and autocrine/paracrine regulator of growth promotion and immuno-stimulation. It controls proliferation, apoptosis, growth and differentiation [32,33]. It is generally believed that GH activity mediated by the cytosolic tyrosine kinase Janus kinase 2 (JAK2) upon GH-GH receptor interaction, resulting in tyrosine phosphorylation of downstream targets including MAP kinases [34]. However, the roles of these pathways, especially that of CDC2, are not well defined. Therefore, it is very important to validate the data obtained from the global proteomics phospho-antibody array, which indicated that the AKT inhibitor prevented the deactivation of CDC2/Cdk1 induced by GH. We stimulated normal growing cells and AKT inhibitor-pre-treated cells, and prepared sub-cellular fractions of the cells at designated time points. The effectiveness of the treatment with the AKT inhibitor was monitored using anti-pAKT (Ser473) antibody (Figure 6B). The results of Western blot using antibodies against p-(Y15)-CDC2/Cdk1 (phosphorylated CDC2/Cdk1 at tyrosine 15) and CDC2/Cdk1 (Figure 6) show that after GH treatment, the phosphorylation of CDC2/Cdk1 (Y-15) accumulated, while the total CDC2/Cdk1 decreased. However, pre-treating cells with the AKT inhibitor reversed the course. Total CDC2/Cdk1 increased and p-(Y15)-CDC2/Cdk1 decreased after GH stimulation. These findings are consistent with the data obtained from the global proteomics phospho-antibody array.

**Figure 7:**
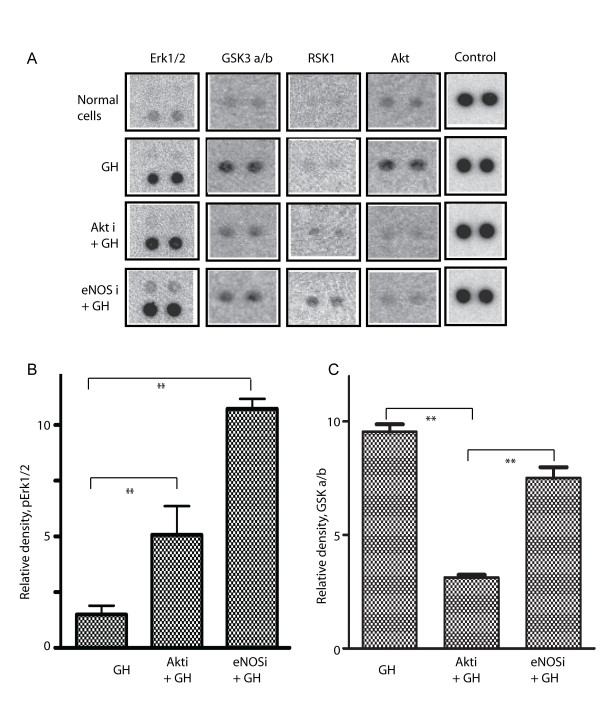
**Proteome Profiler Array™-Human Phospho-MAPK analysis; whole cell extracts from normal cells, and GH challenged (30 minutes) cells, with or without pre-treatment with either AKT or eNOS inhibitors, were analyzed with Proteome Profiler Array™-Human Phospho-MAPK**. Panel A: Data for one representative array. Panel B: Quantitation of phospho-Erk1/2. Panel C: Quantitation of phospho-GSK a/b. n = 2 replicated experiments. *: P < 0.05; **: P < 0.02.

The MAPK/ERK pathway is a signal transduction pathway that couples intracellular responses to the binding of growth factors to cell surface receptors. For the same reason, we further validated the ERK response to GH with or without pre-treatment with the AKT inhibitor. First, using the Proteome Profiler Array™-Human Phospho-MAPK Array (see Methods), we confirmed that the AKT inhibitor and GH have augmentative function, in inducing the phosphorylation of Erk1/2 (Figure 7A, B). Erk1 and Erk2 regulate transcription indirectly by phosphorylating the 90 kDa ribosomal protein S6 kinases (RSKs), a family of broadly expressed Ser/Thr kinases activated in response to mitogenic stimuli, including growth factors and tumor-promoting phorbol esters [35,36], Therefore, it is not unexpected that GH would not increase phosphorylation of RSK1 in normal cells but increases phosphorylation of RSK1 in cells pre-treated with the AKT and eNOS inhibitors.

Another important confirmation of effect of AKT/PKB inhibitor is the significant (P < 0.01) dephosphorylation (activation) of GSK3a/b (glycogen synthase kinase 3), the downstream target of AKT/PKB [37], was induced by the AKT/PKB inhibitor (Figure 7A and 7C). Dephosphorylation of GSK3a/b, however, did not significantly affected by pre-treatment of eNOS inhibitor. These differential effects of AKT/PKB inhibitor and eNOS inhibitor on activation of GSK3a/b are understandable because AKT/PKB is the upstream regulator (inhibitor) of GSK3a/b [38]. In this particular experiment, we also observed that the eNOS inhibitor decreased the ability of GH to induce phosphorylation of AKT. One possible explanation of this effect is that the eNOS inhibitor decreases intracellular NO, thereby decreasing PI-3K and subsequent downstream AKT activities [39,40].

**Figure 8:**
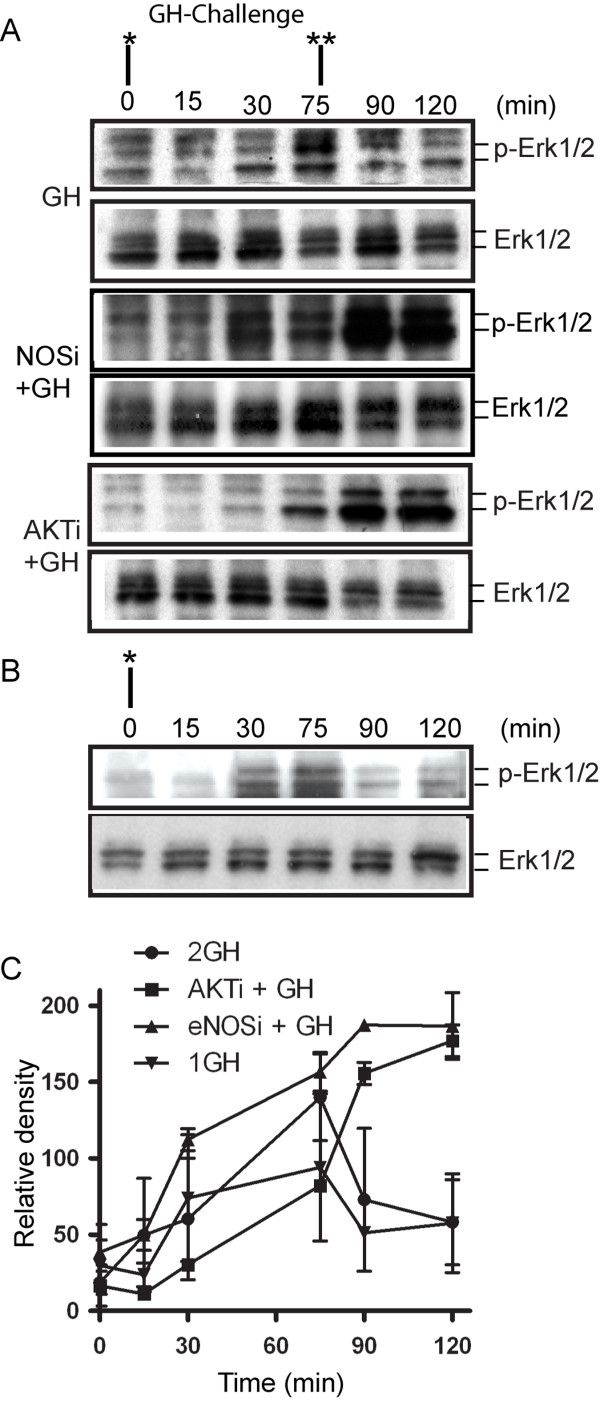
**Both AKT and eNOS inhibitors prolong and augment Erk1/2 phosphorylation**. GH was administrated first at 0 min and a second time at 75 min to cell cultures with or without AKT inhibitor pre-treatment. Whole cell extracts were prepared. A) Western blots using an antibody against phospho-Erk1/2 and Erk1/2 show that the phosphorylation of Erk1/2 starts 15 min after the first GH administration and 2 levels off after 75 min even with secondary GH administration. B) For comparison, cell extracts from cells with single GH stimulation were prepared and Western blots were performed for Erk1/2 and phosphorylated Erk1/2. C) Quantitation of the Western blots data. *: First GH challenge, **: second GH challenge. AKTi: Akt inhibitor, eNOSi: eNOS inhibitor.

We also stimulated normal growing cells and AKT inhibitor-pretreated cells with GH and prepared cell extracts at designated time points with double stimulations of GH. We then analyzed the cell extracts by Western blot using antibodies against Erk1/2 and p-Erk1/2. For the purpose of comparison, the Western blots were first blotted with antibodies against p-ERK. The membranes were then stripped with Restore PLUS Western Blot Stripping Buffer (Pierce Biotechnology, now Thermo Scientific) and re-blotted with anti-ERK. The Western blots (Figure 8A and 8B) are quantitated and presented in Figure 8C. After GH treatment, phospho-Erk1/2 levels increased, peaked at about 30 to 60 min and levelled off, suggesting that the biochemical events associated with the initiation of the GH signal transduction cascade did in fact occur (Figure 8A, B). However, the second GH dosage added to the cell culture did not induce more phosphorylation in Erk1/2 as comparison to the phosphorylation pattern of Erk1/2 from cells with single stimulation of GH (Figure 8B and 8C). In cells pre-treated with the AKT inhibitor, the levels of phospho-Erk1/2 increased, peaked at much later time points, and secondary GH enhanced the phosphorylation of Erk1/2. The phosphorylation of Erk1/2 in eNOS-inhibitor-pre-treated cells shows similar patterns after GH stimulation to AKT inhibitor-pre-treated cells (Figure 8A and 8C).

## Discussion

Extensive research over the past decades on the wiring of signal transduction pathways has uncovered a level of complexity. It is clear now that individual pathways are only small parts of fully integrated networks within the cell and there is no signal pathway operating in isolation [7]. In the present study, we used an established bovine cell line (MDBK) to determine the roles of AKT/PKB and eNOS, as well as the downstream elements in the GH signaling pathway. Using a specific AKT/PKB inhibitor and the functional proteomic approach, we were able to detect the activities of multiple signal transduction pathway elements, the downstream targets of the AKT/PKB pathway and the modification of those responses by treatment with GH. Three approaches were applied, and separate and independent *in vitro *experiments were conducted to define the potential mechanism of AKT/eNOS activities. The pre-treatment of MDBK cells with the AKT inhibitor prior to the addition of media containing GH resulted in prolonged and augmented Erk1/2 phosphorylation (activation) after the GH challenge. AKT inhibitor also antagonizes the effects of GH, which induces deactivation (phosphorylation) of CDC2/Cdk1. Inhibiting the AKT/PKB activity reduced or eliminated the activation (phosphorylation) of eNOS. Based on these observations, we conclude that the AKT/eNOS signaling module may function as a potential feedback loop in the GH signal pathway.

Endothelial NOS was first identified as a substrate of AKT/PKB [28]. Stimulation of Phosphoinositide 3-kinases (PI3K)/AKT by shear stresses and VEGF (vascular endothelial growth factor) elicits the serine phosphorylation of eNOS, thereby enhancing enzyme activity in a Ca2+-independent manner. The activation of eNOS by AKT contributes to the important physiological effects of AKT on apoptosis, cell attachment and cell proliferation [41-45]. In fact, serine/threonine kinase AKT/PKB is regarded as a central node in cell signaling downstream of the growth factor, cytokines, and other cellular stimuli [[7] and references wherein]. The apparent responsiveness of cells to the single or double GH challenge was either prolonged or augmented, assuming that the measured level of phospho-eNOS and CDC2/Cdk1 is indicative of maintained responsiveness. However, the downstream pathways of AKT/PKB which modulate its effects are still undefined. The results reported here indicate that AKT/PKB can modulate the effects of GH on cell growth and survival either directly or indirectly through the MAPK- and CDC2/Cdk1-dependent signaling cascade.

The CDC2/Cdk1 protein is a member of the Ser/Thr protein kinase family. This protein is a catalytic subunit of the highly conserved protein kinase complex known as the M-phase promoting factor (MPF), which is essential for the G1/S and G2/M phase transitions of the eukaryotic cell cycle. Mitotic cyclins stably associate with this protein and function as regulatory subunits. As the effect of the AKT inhibitor, CDC2 maintains its dephosphorylation (activation) status. It has been reported that CDC2/Cdk1 and kinase from the tyrosine kinase family are involved in the GH response. Inhibitors of tyrosine kinase prevented GH effects on IGF-1 release [46] and apoptosis [47] in porcine granulosa cells. The anti-apoptosis effect of GH on porcine granulosa cells can also be prevented by inhibitors of protein kinase G and CDC2/Cdk1 kinases, suggesting that these kinases are required for GH mediation [48]. Phosphorylation of CDC2/Cdk1 on Thr14 (mainly by Myt1 kinase) and Tyr15 (mainly by Wee 1 kinase) inhibits its activity during the G2 phase of the cell cycle, presumably through a direct effect on the phosphotransfer activity, while dephosphorylation of CDC2/Cdk1 on both sites by the phosphatase (CDC25) of CDC2/Cdk1 de-inhibits CDC2/Cdk1 during early mitosis [49]. The regulation of the spatiotemporal pattern of Cdk1/cyclin B activity is pivotal for the normal cell cycle and is subject to multiple control steps [50]. Our approaches, both the global proteomics phospho-antibody array and Western blot analysis of the kinetic changes of the activity status of CDC2/Cdk1 responding to GH stimulation, have similar and consistent results. Pre-treatment of MDBK cells with the AKT inhibitor results in antagonized GH effect and reduced CDC2/Cdk1 phosphorylation after the GH challenge, indicating the involvement of CDC2/Cdk1 in the GH response pathway. The activity of CDC2/Cdk1 is modulated, directly or indirectly, through the AKT/PKB pathway.

Another important finding of the present study is that the AKT inhibitor also induces the prolonged and augmented activation of Erk1/2, induced by the GH challenge. The Ras/Raf/MEK/Erk and PI3K/PTEN/AKT signaling cascades play critical roles in the transmission of signals from growth factor receptors to regulate gene expression and prevent apoptosis [51]. The Raf/MEK/Erk kinase cascade is pivotal in transmitting signals from membrane receptors to transcription factors that control gene expression, culminating in the regulation of cell cycle progression. This cascade can prevent cell death through Erk2 and p90 (Rsk) activation and the phosphorylation of apoptotic and cell cycle regulatory proteins. The PI3K/AKT kinase cascade also controls apoptosis and can phosphorylate many apoptotic and cell cycle regulatory proteins. Activated ERK regulates growth factor-responsive targets in the cytosol and also translocates to the nucleus where it phosphorylates a number of transcription factors that regulate gene expression. These pathways are interwoven, as AKT can phosphorylate Raf and result in its inactivation, and Raf can be required for the antiapoptotic effects of AKT [52]. The individual effects of activated Raf and AKT on proliferation, apoptosis and autocrine growth factor synthesis have been studied [52]. Activation of either Raf or AKT hindered cell death. It has been established that AKT blocks Erk signaling through the inhibition of Raf1 [53,54]. AKT/PKB can directly phosphorylate Raf1 on T259, and this can lead to an inhibitory effect of AKT on the Erk pathway [54]. Our finding reported here indicates that the ERK signaling pathway is directly involved in the GH response as reported by many laboratories [16,25,55-57]. AKT/PKB modulates this response and may exert its modulating function through crosstalk with the ERK pathway. Because AKT activation also leads to many downstream signals other than eNOS, it is important to further define the role of crosstalk between the AKT/PKB-eNOS pathway and other signaling pathways or the elements of those pathways such as JNK1, TYH, Erb2, elF-4E and Elf2a. Although this is beyond the immediate scope of the present research, such a study may reveal that the AKT-associated modulating of Erk and eNOS phosphorylation participates in what has been characterized as GH-induced GH desensitization.

## Conclusion

The major conclusions of this study are: We found the MAP kinase and CDC2 kinase-dependent intracellular mechanisms are involved in or are the targets of the GH's action processes. These activities are probably directly or indirectly modulated by AKT/PKB pathways. We propose that the AKT/PKB-eNOS module likely functions as a negative feedback mediator of GH actions.

## Methods

### Cell culture and treatment

Madin-Darby bovine kidney epithelial cells (MDBK, American Type Culture Collection, Manassas, VA., and Catalog No. CCL-22) were cultured in Eagle's minimal essential medium supplemented with 5% fetal bovine serum (Invitrogen, Carlsbad, CA) in a 25 cm^2 ^flask with medium renewal twice per week. Cell cultures were maintained in a water-jacked incubator with 5% CO_2 _at 37°C. Sub-cultivations were performed when cells attained 80 to 90% confluence, according to the product information supplied by American Type Culture Collection. Cells were used for treatment testing at approximately 50% confluence during the exponential phase of growth. Each experiment was replicated in three independent experiments with identical conditions unless indicated otherwise.

### *In vitro *GH challenge and modulation of the eNOS/AKT axis

Cells were incubated in fresh medium without fetal bovine serum for about 6 h before the treatments. No BSA or other protein was added during serum starvation. The serum-free medium was aspirated from the T-25 flasks and replaced with the same type of medium containing the recombinant GH. GH (recombinant bovine GH, Monsanto Inc., St. Louis, MO) was added to the culture at 200 ng/ml final concentration. The dose of 200 ng/ml was chosen based on a previous report [16] and our dose response experiment on the MDBL cells (data not shown). For the AKT and eNOS inhibition experiments, 10 μM AKT inhibitor (Catalog # 124005, CalBiochem, San Diego, CA.) or eNOS inhibitor (1 mM N^G^-Monomethyl-Λ-arginine, CalBiochem) were added to the culture media 30 min before GH treatment. For time course studies, the activity of cells cultured in T-25 flasks was arrested with the addition of M-Per^® ^buffer and gentle agitation (Pierce, Inc., Rockford, IL, as per manufacturer's instructions) at time '0' (with the addition of fresh serum – GH-free medium) and at designated time points (as indicated in each experiments and related figures) after the addition of GH. As different experimental protocols dictated, dispersed cells and contents were sonicated to a uniform consistency and the protein content was measured by the traditional Lowry procedure. M-PER is a non-denaturing detergent-based protein extraction buffer used to extract total cellular protein quickly. A protease inhibitor cocktail for use with mammalian cell extracts, and phosphatase inhibitor cocktails I and II (Sigma, St. Louis, MO), were added in M-PER immediately before use. For the experiments including a second GH challenge at 75 min, the medium containing GH was aspirated and replaced with fresh GH-containing medium. The 75 min samples were taken immediately after the second GH challenge.

### Subcellular extraction and localization of protein from MDBK cells

Compartmental localization of the proteins was assessed by the subcellular fractionation of cultured cells. After treatment with GH at the indicated time points, proteins were extracted from MDBK cells using the ProteoExtract^® ^subcellular extraction kit (Calbiochem), following the manufacturer's instruction. Stepwise extraction resulted in the separation of proteins into cytosol, membrane and nuclear fractions. GAPDH and core histone content were used as markers for the cytosal and nuclear fractions, respectively.

### Cellular protein separation and Western analysis

Cellular proteins were separated for identification by standard SDS-polyacrylamide gel electrophoresis (SDS-PAGE) using 4% to 20% gradient Tris-glycine gels (Invitrogen, Carlsbad, CA) under the reducing conditions suggested by the manufacturer. Pre-stained molecular weight standards (SeeBlue-plus2, Invitrogen) were included. The separated proteins were eluted from the gels and transferred to pure nitrocellulose membranes (Protran, 0.2 μm, Schleicher & Schuell, Dassel, Germany) by semi-moist electro-transfer. Nonspecific binding of the reagent antibodies to the nitrocellulose matrix was blocked by incubating the membranes with 5% fat-free dry milk in PBS for 1 h prior to incubation with the antibodies specific to each desired protein (1:1000 diluted in PBS plus 0.1% Triton 100). Western blot analyses were performed using anti-AKT/PKB, anti-phospho-AKT/PKB (Cell Signaling Technology, Beverly, MA), anti-endothelial NOS (eNOS), anti-phospho-eNOS (1177phosphoserine-eNOS) (Upstate Biotech Inc., Lake Placid, NY), anti-CDC2/Cdki1 (Abcam) and p-(Y-15) CDC2/Cdk1(R & D Systems) antibodies. A secondary antibody (Immuno-Pure, anti-mouse-IgG or anti-rabbit-IgG conjugated with horseradish peroxidase, Pierce Biotechnology, 1:25000 diluted with PBS) was added and incubated for1 h. Membranes were washed 5 times with PBS and 0.1% Triton 100. Immunoblots were exposed to SuperSignal West Pico Stable Peroxide solution with luminol/enhancer (Pierce Biotechnology) according to the manufacturer's instructions. Western blots were then scanned and analyzed using UN-SCAN-IT software (Silk Scientific, Orem, Utah) to quantify the density of the bands. The quantification was done with exposures that are in the linear range. Protein gels were stained with SimpleBlue™ (Invitrogen) to visualize the proteins and used to normalize the Western blots. Data were statistically analyzed by ANOVA using GraphPad Prism 5.00 for Windows (GraphPad Software, San Diego, CA, USA). When significant effects were detected (P < 0.05), differences between means were estimated by Tukey's multiple comparison test. Tukey's test is a statistical test generally used in conjunction with an ANOVA to find which means are significantly different from one another. The test compares the means of every treatment to the means of every other treatment, and identifies where the difference between two means is greater than the standard error would be expected to allow. Data are presented as means +/- SEM.

### Functional proteomic analysis

The global proteomics phosphoantibody array-based approach was performed to analyze the signal transduction pathways of the GH axis and the roles of AKT/PKB in MDBK cells utilizing the Kinetworks Phospho-Site Screen (KPSS)-6.0 (Kinexus Bioinformatics Corporation, Vancouver, Canada). A wide range of phosphorylation site-specific antibodies were used in a quantitative fashion as a specific assay for the regulation of diverse cell signaling pathways . KPSS-6.0 was used to screen and quantitate the phosphorylation status of 33 different phospho-sites from over 150 known phospho-sites in kinase substrates, in phospho-proteins with antibodies that recognize specific phosphorylated epitopes of target proteins. A total of 500 μg of whole cell lysates from each experimental cell group, control cells (without GH) and GH treated cells (with or without AKT inhibitor pre-treatment) was used for each KPSS phosphoantibody array screen. This is an antibody-based method (Kinetworks) that relies on Sodium dodecyl sulphate (SDS)-polyacrylamide minigel electrophoresis and multilane immunoblotters to permit the specific and quantitative detection of numerous protein kinases or other signal transduction proteins simultaneously [58]. To further investigate the phosphorylation of the kinases in the mitogen-activated protein kinase (MAPK) pathway, we also used the Proteome Profiler Array™-Human Phospho-MAPK Array (R & D Systems, Inc). Sample preparation, reagent preparation and array protocol followed the manufacturer's instruction.

## List of abbreviations used

AKT/PKB: Protein kinase B; eNOS: endothelial nitric oxide synthase; CDC2/Cdk1: Cell division control protein 2/Cyclin-dependent protein kinase 1); GH: growth hormone; JAK2: Janus kinase 2; MDBK: Madin-Darby bovine kidney epithelial cells; JNK1: cJun NH2 terminal kinase 1; Erk: extracellular signal-regulated kinases; GSK3a/b: glycogen synthase kinase 3.

## Competing interests

The authors declare that they have no competing interests.

## Authors' contributions

CJL conceived the project and developed the conception of the study, designed and coordinated the study, performed the experiment and prepared manuscript, THE and SK participated in the conception of the study, performed parts of the experiments and helped to prepare the manuscript. All authors read and approved the final manuscript.
